# Prelacteal feeding and associated factors in Ethiopia: systematic review and meta-analysis

**DOI:** 10.1186/s13006-018-0193-6

**Published:** 2018-11-28

**Authors:** Habtamu Temesgen, Ayenew Negesse, Wubetu Woyraw, Temesgen Getaneh, Molla Yigizaw

**Affiliations:** 1 Department of Human Nutrition and Food Sciences, College of Health Science, Debre Markos University, Debre Markos, Ethiopia; 2 Department of Midwifery, College of Health Science, Debre Markos University, Debre Markos, Ethiopia; 3 Department of Public Health, College of Health Science, Debre Markos University, Debre Markos, Ethiopia

**Keywords:** Prelacteal feeding, Pooled prevalence, Ethiopia, Associated factors

## Abstract

**Background:**

Prelacteal feeding can be defined as giving any solid or liquid foods other than breast milk during the first three days after birth. It affects timely initiation of breastfeeding and exclusive breastfeeding practices. Even though the issue was investigated in Ethiopia, fragmented and inconsistent findings were reported. Therefore, the main objective of this meta-analysis was to estimate the pooled prevalence of prelacteal feeding and associated factors in Ethiopia.

**Methods:**

The preferred reporting items for systematic reviews and meta-analyses guideline was followed. Articles were systematically searched through different searching mechanisms. Joanna Briggs Institute Meta-Analysis of Statistics Assessment and Review Instrument adapted for cross-sectional study design was used for quality assessment of each individual study. The total of 28 studies were included and analyzed. The random effect model was used to estimate the pooled prevalence; subgroup analysis and meta-regression were performed to identify the probable source of heterogeneity. Both Egger’s, and Begg’s test were used to check publication bias. The effects between associated factor variables, and prelacteal feeding practices were tested.

**Results:**

A total of 492 studies were retrieved and 28 studies were included in the meta-analysis. The pooled prevalence of prelacteal feeding practice in Ethiopia was 25.29% (95% Confidence Interval [CI] 17.43, 33.15) with severe heterogeneity (I^2^ = 99.7, *p* < 0.001) and no publication bias. Antenatal care (Odds Ratio [OR] 0.25, 95% CI 0.09, 0.69), counselling on infant feeding (OR 0.37, 95% CI 0.22, 0.63), timely initiation of breastfeeding (OR 0.28, 95% CI 0.21, 0.38) and an urban residence (OR 0.47, 95% CI 0.26, 0.86) had lower odds, while home birth had higher odds (OR 3.93, 95% CI 2.17, 7.10) of prelacteal feeding in Ethiopia.

**Conclusions:**

In Ethiopia, one in four children were given prelacteal foods. Mothers who gave birth at home are more prone to give prelacteal foods. Whereas, antenatal care, timely initiation of breastfeeding, counseling on infant feeding and an urban residence decreases prelacteal feeding practices in Ethiopia. Therefore, the government and health institutions should focus to increase maternal health service utilization and promote infant and young child feeding practices according to the guideline.

## Background

Prelacteal feeding is giving any solid or liquid foods other than breast milk during the first 3 days after birth [1–3]. Even though the World Health Organization (WHO) recommends exclusive breastfeeding (EBF) for the first 6 months, 823,000 children under 5 years of age, annually, were suffering from improper breastfeeding practice including prelacteal feeding [4, 5] and every day, 3000 up to 4000 infants die in the developing world from diarrhea and acute respiratory infections [6, 7].

Prelacteal feeding affects the timely initiation of breastfeeding and exclusive breastfeeding [3]. Globally, suboptimal infant feeding, including prelacteal feeding contributes 45% of neonatal mortality, 30% of diarrheal mortality and 18% of acute respiratory deaths [8, 9]. Prelacteal feeding reduces the immunological benefits that gains from colostrum and increases the risk of susceptibility to infection [10]. Furthermore, directly it predisposes newborns to pathogenic contaminants creates physiological disruptions in the immature gastrointestinal system and discourages newborns from initiating breastfeeding. In addition, mother-baby bonding may be interrupted and interfering with breast milk production [10–13].

Prelacteal feeding practice is a predominant problem in the developing world. Prelacteal feeding practice in Vietnam and India was 73.3 and 40.1% respectively [10, 14]. A study in Africa revealed that about 32·2% in Sub-Saharan [15], 60% in Egypt [16], 31.3% in Uganda [17] of mothers practiced prelacteal feeding. Data from the Ethiopian Demographic Health Survey (EDHS) of 2011 report showed that 27% of infants were given prelacteal feedings within the first 3 days of life [18]. Also, the national survey revealed that prelacteal feeding was 28.9% in Ethiopia [19].

In Ethiopian, different independent and fragmented studies have been conducted to assess the magnitude of prelacteal feeding practice and its determinants. These discrete studies reported that the magnitude of prelacteal feeding in Ethiopian were ranging from 5.9% up to 75.8% [20, 21]. Prelacteal feeding practice is a well-documented phenomenon and need to be the focus area of research to determine the prevalence and its predictors in Ethiopia. A number of researchers have reported the prevalence of prelacteal feeding practices in Ethiopia [7, 9, 13, 20–43].

Those individual studies indicated that there is great variation and inconsistencies of prelacteal feeding and predictors in Ethiopia. The common factors reported by the above studies were place of residence, place of delivery, antenatal care, counselling on feeding and time to initiate breastfeeding [7, 9, 13, 20, 22–25, 27, 28, 30, 34, 41]. The reasons for disparity of prelacteal feeding practice and its predictors in Ethiopia have not yet been investigated. In addition to this gap, there are no documented data on pooled prevalence of the prelacteal feeding practice in Ethiopia. Therefore, the main objective of this systematic review and meta-analysis was to estimate the pooled prevalence of prelacteal feeding practice and its associated factors in Ethiopia.

The findings of this study will be an input to policy makers and program planners of the Ethiopian government to design appropriate interventions to decrease prelacteal feeding practice and also important to intervene important predictors to reduce prelacteal feeding practices. This review also will give the national figure for future researchers.

## Methods

### Searching strategies

This systemic review and meta-analysis were designed to estimate the pooled prevalence of prelacteal feeding and its associated factors of prelacteal feeding in Ethiopia. Initially meta-analysis and systematic reviews, including registered protocols were searched to avoid duplications. It confirmed that there was no review and meta-analysis conducted related to prelacteal feeding in Ethiopia. Published research reports of prelacteal feeding and its associated factors were searched. We systematically reviewed and analyzed published research articles to determine the pooled prevalence of the prelacteal feeding practice and its factors in Ethiopia. To identify published articles, major databases PUBMED/MEDLINE, Cochrane library, Google and Google Scholar were used. In addition, reference lists were used. The key term used in PubMed search was “prevalence” OR “magnitude” AND “prelacteal” AND “feeding practice” AND “Ethiopia” AND “associated factors” AND “age less than five years”. The search was conducted from May, 2018 to June 30, 2018.We followed the Preferred Reporting Items for Systematic Reviews and Meta-Analyses (PRISMA) guideline during the systematic review [44].

### Inclusion criteria

#### Study scope

All studies which report the prevalence of prelacteal feeding and associated factors of prelacteal feeding in Ethiopia were included under this systematic review and meta-analysis.

#### Study design

Cross-sectional study design was included.

#### Language

Articles published in the English language were included.

#### Population

All studies conducted in Ethiopia were considered.

#### Publication and publication year

Published articles until June/ 2018 were included.

### Exclusion criteria

Articles published other than in English language and studies which didn’t report specific outcomes for prelacteal feeding were excluded.

### Data abstraction

The database search results were collected and duplicate articles were removed manually using endnote (version X7). Data were extracted by two authors using a standardized data extraction spread sheet. Data extraction sheet included study characteristics such as: (1) Authors’ name, region, study year, publication year, study design, study setting, sample size, response rate, studies’ quality score and sampling; (2) prevalence of prelacteal feeding; (3) residence, antenatal care, place of delivery, initiation of breastfeeding within 1 hours were also extracted from each individual study. Those categorical variables tabulated (a, b, c and d) with prelacteal feeding during abstraction.

### Quality assessment (appraisal) of studies

The database search results were combined and duplicate articles were removed manually using endnote (version X7). Joanna Briggs Institute Meta-Analysis of Statistics Assessment and Review Instrument (JBI-MAStARI) adapted for both cross-sectional/case-control study design was used [45]. Three independent reviewers critically evaluated each individual paper. Discrepancies between those reviewers were solved by discussion. If not, a third reviewer was involved to resolve inconsistencies in between the two independent reviewers. Studies, which score five and above from a total of nine scores were included in the final systematic review and meta-analysis.

### Outcome measurements

This review and meta-analysis have two main outcomes. The primary outcome was prevalence of prelacteal feeding practices. The second outcome was factors associated with prelacteal feeding practices in Ethiopia.

### Data analysis

The extracted data were entered into an excel sheet and imported to STATA version 14 for analysis. Heterogeneity among reported prevalence was assessed by using the inverse variance (I^2^) with Cochran Q statistic of 25, 50 and 75% as low, moderate and sever heterogeneity respectively with *p* - value less than 0.05 [46]. Random effects meta-analysis model was used to estimate the pooled prevalence of prelacteal feeding. The forest plot was also used to visualize the presence of heterogeneity graphically. Possible differences between the studies were explored by subgroup analyses and meta-regression. The finding was presented using forest plot with respective odds ratio and 95% confidence intervals. Evidence of publication bias was assessed using both Egger’s, and Begg’s test with *p* - value of less than 0.05 as a cutoff point to declare the presence of publication bias [45, 47]. For the second outcomes, pooled odds ratios with 95% CI for each factor were used to determine the association between prelacteal feeding practices and its factors (antenatal care, place of delivery, timely initiation of breastfeeding, counselling on infant feeding and place of residences).

## Results

### Selection of studies

**Fig. 1:**
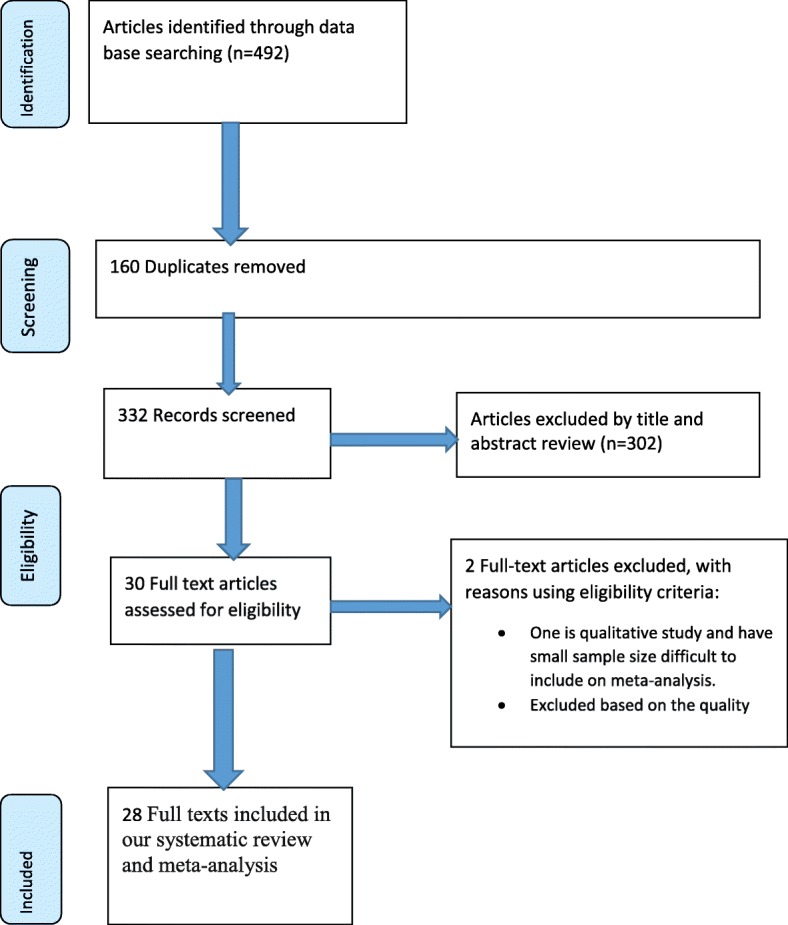
PRISMA flow diagram of included studies to estimate the pooled prevalence of prelacteal feeding practices and its predictors in Ethiopia

A total of 492 articles searched through the electronic searches of which 160 duplicated articles were excluded. From the remaining 332 articles, 302 articles were excluded after reading of titles and abstracts. Finally, 30 full text articles were accessed for eligibility criteria. Based on the predefined criteria and after critical appraisal (two articles were excluded [48, 49]), 28 articles were included in the final analysis (Fig. 1).

### Characteristics of included studies

The total of twenty-eight articles was included in this meta-analysis and systematic reviews that met the inclusion criteria. All the included studies were published from 2011 up to 2018. All included studies used cross-sectional study design. A total of 28,435 mothers participated in these studies using an estimated sample size range from 184 [38] up to 6761 [34] to estimate the pooled prevalence prelacteal feeding practice and its associated factors in Ethiopia.

From the total of 28 articles, 15 studies were conducted in Amhara regional state [13, 21, 22, 24, 27–29, 32–34, 36, 37, 41–43]; three studies at South Nations Nationalities and Peoples of Ethiopia national regional state (SNNP) [9, 30, 38]; five studies at Oromia national regional state [7, 20, 35, 39, 40]; two studies at Tigray national regional state [50, 51]; one study at afar regional state [25]; one study at Binishangul gumuz regional state [31] and one study was conducted at national level in Ethiopia [23]. Twenty-five studies were conducted in the community and the rest three were conducted at the institution based. The results were tabulated according to the prevalence of prelacteal feeding (Table 1).

**Table 1:** Characteristics of 28 included studies to estimate the pooled prevalence of prelacteal feeding practices and its associated factors in Ethiopia

Id	Authors	Region	Study years	Publication years	Study design	Study setting	Sampling method	Sample size	Prevalence (%)
1	Hailemariam et al. [20]	Oromia	2014	2015	Cross-sectional	Community	Multistage	593	5.9
2	Zegeye Abebe et al. [42]	Amhara	2014	2017	Cross-sectional	Community	Multistage	707	8.2
3	Adugna [30]	SPNN	2012	2014	Cross-sectional	Community	Simple random	383	8.9
4	Belachew et al. [23]	Ethiopia	2015	2016	Cross-sectional	Community	Multistage	7692	8.92
5	Bayissa et al. [40]	Oromia	2014	2015	Cross-sectional	Community	Simple random	371	9.7
6	Bililign et al. [13]	Amhara	2015	2016	cross-sectional	Community	Multistage	782	11.1
7	Bimerew et al. [32]	Amhara	2015	2016	Cross-sectional	Community	Multistage	739	11.8
8	Asfaw et al. [39]	Oromia	2013	2015	Cross-sectional	Community	Multistage	778	12.1
9	Teka et al. [50]	Tigray	2013	2015	Cross-sectional	Community	Multistage	530	12.8
10	Asemahagn [43]	Amhara	2014	2016	Cross-sectional	Community	Census	332	15
11	Demilew et al. [33]	Amhara	2016	2017	Cross-sectional	Community	Simple random	412	15
12	Ayana et al. [31]	B/Gumuz	2015	2017	Cross-sectional	Community	Systematic random	761	15.9
13	Haile et al. [38]	SPNN	2012	2015	Cross-sectional	Institution	Multistage	184	16.8
14	Tilahun et al. [41]	Amhara	2013	2016	Cross-sectional	Community	Simple random	409	16.8
15	Alemayehu et al. [51]	Tigray	2013	2014	Cross-sectional	Community	Simple random	418	17.2
16	Gualu et al. [24]	Amhara	2016	2017	Cross-sectional	Community	Census	262	19.1
17	Yenit et al. [28]	Amhara	2016	2017	Cross-sectional	Institution	Census	367	19.1
18	Genetu et al. [37]	Amhara	2016	2017	Cross-sectional	Community	Systematic random	367	19.1
19	Tilahun Tewabe [29]	Amhara	2015	2016	Cross-sectional	Community	Simple random	405	20.2
20	Chea and Asefa [9]	SPNN	2016	2018	Cross-sectional	Community	Multistage	597	25.5
21	Tariku et al. [27]	Amhara	2015	2016	Cross-sectional	Community	Simple random	822	26.8
22	Legesse et al. [22]	Amhara	2014	2014	Cross-sectional	Community	Systematic random	630	38
23	Liben et al. [25]	Afar	2016	2017	Cross-sectional	Community	Multistage	615	42.9
24	Bekele et al. [7]	Oromia	2013	2014	Cross-sectional	Institution	Systematic random	612	45.4
25	Derso et al. [34]	Amhara	2014	2017	Cross-sectional	Community	Census	6761	56
26	Fentahun et al. [36]	Amhara	2014	2016	Cross-sectional	Community	Multistage	633	58.7
27	Egata et al. [35]	Oromia	2011	2013	Cross-sectional	Community	Simple random	860	75.8
28	Mekuria and Edris [21]	Amhara	2013	2015	Cross-sectional	Community	Simple random	413	75.8

### Pooled prevalence of prelacteal feeding practice in Ethiopia (Meta-analysis)

**Fig. 2:**
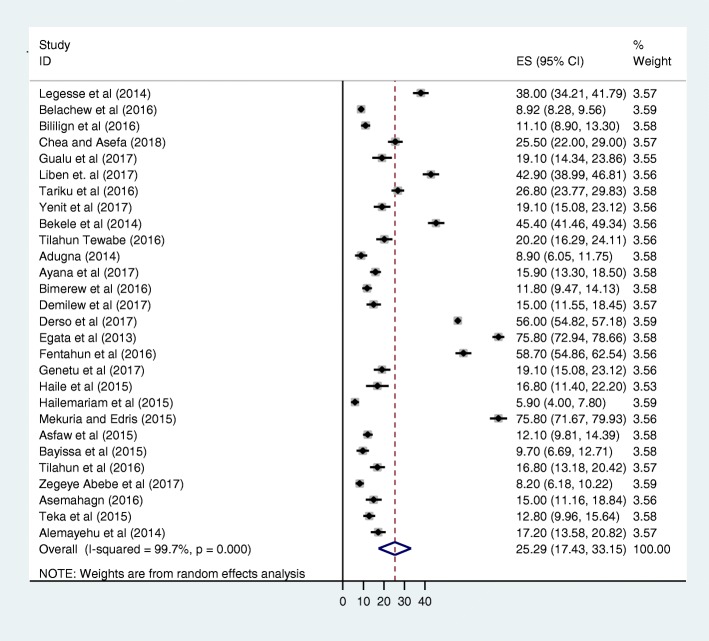
Forest plots showing the pooled prevalence of prelacteal feeding practice in Ethiopia

The pooled prevalence of prelacteal feeding practice in Ethiopia was 25.29% (95% CI 17.43, 33.15) (Fig. 2). As shown in the forest plot below, statistically significant heterogeneity was identified (I^2^ = 99.7%; *p* < 0.001) indicating that the use of random effects models for estimating the pooled estimates is applicable. The significant magnitude of the heterogeneity also suggests the need to conduct subgroup analysis to identify the sources of heterogeneity (Fig. 2).

### Subgroup analysis

Subgroup analysis was done based on study area (regions), study years, sampling techniques and study setting to identify the possible source of heterogeneity across studies (Table 2). The subgroup analysis result directed that the source of heterogeneity was not due to the study area, study years, sampling techniques and study setting (*p* < 0.001) (Table 2).

**Table 2:** Subgroup analysis which indicates the pooled prevalence prelacteal feeding practices in Ethiopia

Subgroups		Number of studies included	Prevalence (95% CI)	Heterogeneity statistics	*p -* value	I^2^	Tau-squared
Region	Amhara	15	27.37 (15.50, 39.25)	3764.65	< 0.001	99.6%	547.17
	Oromia	5	29.76 (4.04, 55.49)	1904.20	< 0.001	99.8%	859.03
	SNNP	3	17.04 (5.97, 28.11)	52.18	< 0.001	96.2%	91.43
	^a^Others	5	19.43 (10.17, 28.69)	319.43	< 0.001	98.7%	109.31
Time of study years	Before 2015	15	30.94 (17.51, 44.37)	5314.10	< 0.001	99.7%	701.28
	2015 and above	13	18.70 (14.10, 23.30)	543.91	< 0.001	97.8%	68.67
Study setting	Community based	25	25.07 (16.67, 33.48)	8171.93	< 0.001	99.7%	456.90
	Institution based	3	27.15 (8.37, 45.92)	109.26	< 0.001	98.2%	269.91
	Systematic random sampling	4	29.57 (15.19, 43.94)	200.50	< 0.001	98.5%	211.74
Sampling techniques	Multistage random sampling	11	19.38 (13.10, 25.66)	999.06	< 0.001	99.0%	110.54
	Simple random sampling	9	29.57 (11.93, 47.22)	2037.82	< 0.001	99.6%	726.33
	Census	4	27.34 (1.61, 53.08)	798.42	< 0.001	99.6%	686.01

^a^Tigray, B/gumuz, Afar and national study

The lowest pooled prevalence of prelacteal feeding practice was indicated in SPNN 17.04% (95% CI 5.97, 28.11) and the highest was in Oromia region 29.76% (CI 4.04, 55.49) followed by Amhara region 27.37% (95% CI 15.50, 39.25). There was decrement of prelacteal feeding practices starting from 2015 of 18.70% (95% CI 14.10, 23.30) (Table 2). In addition to subgroup analysis, publication bias as the source of heterogeneity was also checked using both Begg’s and Egger’s test. The result of Begg and Egger tests were not identified as the source of heterogeneity pooled prevalence of prelacteal feeding practices at *p* - value of (*p* = 0.055) and (*p* = 0.181) respectively.

### Meta regression

Besides subgroup analysis and publication bias, meta regression was also assumed by considering both continuous and categorical data to identify associated factors of heterogeneity for the pooled prevalence of prelacteal feeding practices. Sample size, study year, study setting and sampling techniques were considered in the meta-regression. However, the meta-regression indicated that the pooled prevalence of prelacteal feeding was not associated with study year, sample size, study setting and sampling techniques (Table 3).

**Table 3:** Meta regression to identify source of heterogeneity for the prevalence of prelacteal feeding practices in Ethiopia

Variables		No	Coefficients	*p* - value
Study year	2015 or later	13	Reference	Reference
	Before 2015	15	12.15842	0.111
Sample	Sample size	28	0.0011158	0.617
Study setting	Community based	25	−2.059248	0.871
	Institution based	3	Reference	Reference
Sampling techniques	Censes	4	Reference	Reference
	Multistage	11	12.05831	0.519
	Simple random	9	12.41248	0.861
	Systematic random	4	14.6074	0.881

### Associated factors of prelacteal feeding practices in Ethiopia

The overall pooled odds ratio was estimated for different factors reported repeatedly that affect the prelacteal feeding practices in Ethiopia. Antenatal care for index child [7, 22, 24, 28], place of delivery [7, 9, 13, 22–24, 27, 28], counselling about feeding [9, 13, 24, 25, 28], time to initiation of breastfeeding [7, 13, 20, 22–25, 29, 30, 34, 41] and place of residence [7, 22, 23, 28] were repeatedly reported as significant factors for prelacteal feeding practices.

**Fig. 3:**
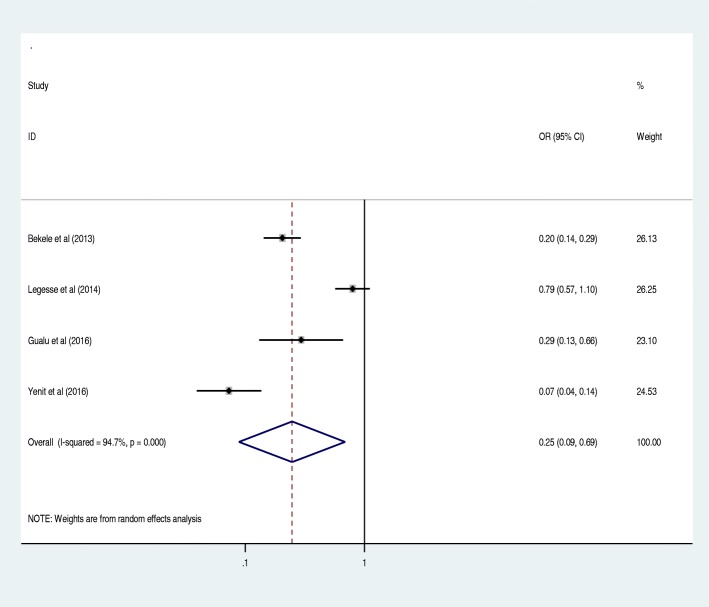
The pooled odds ratio of the association between antenatal care and prelacteal feeding in Ethiopia

**Fig. 4:**
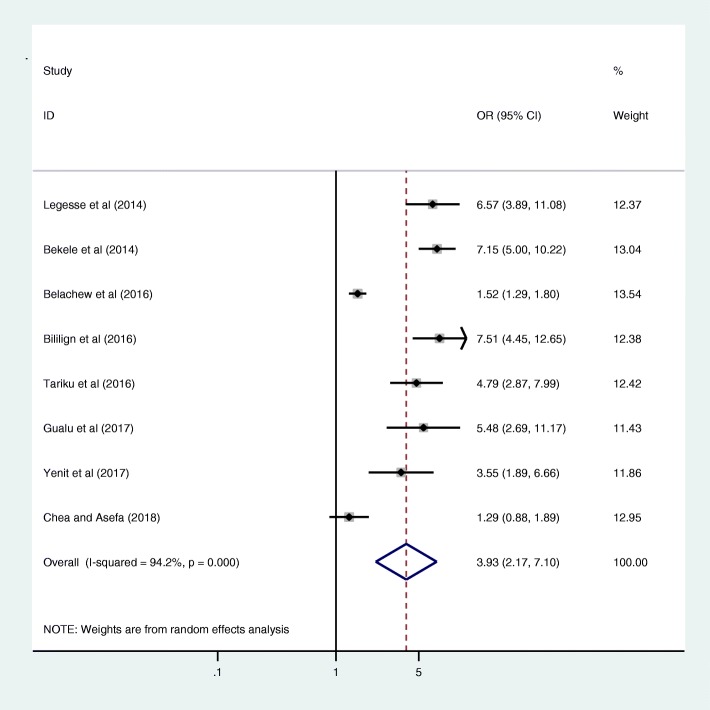
The pooled odds ratio of the association between place of delivery and prelacteal feeding

**Fig. 5:**
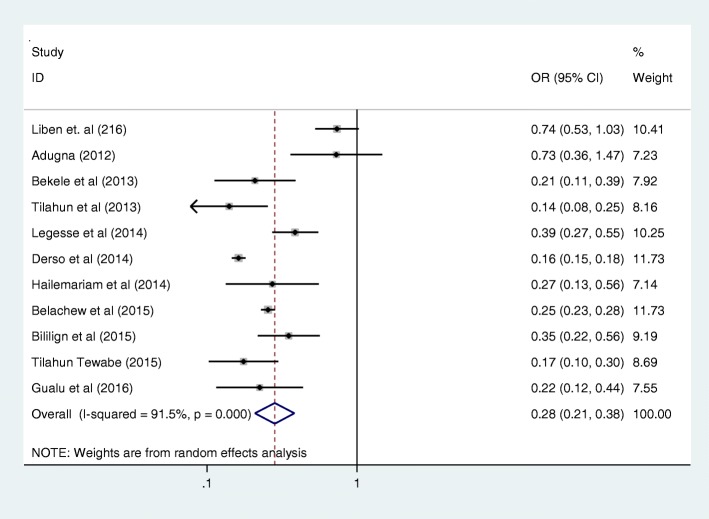
The pooled odds ratio of the association between counselling on infant feeding and prelacteal feeding

**Fig. 6:**
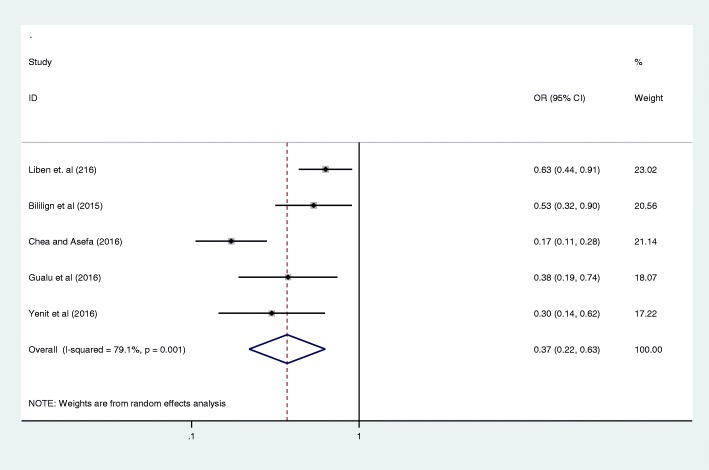
The pooled odds ratio of the association between time to initiation of breastfeeding and prelacteal feeding

**Fig. 7:**
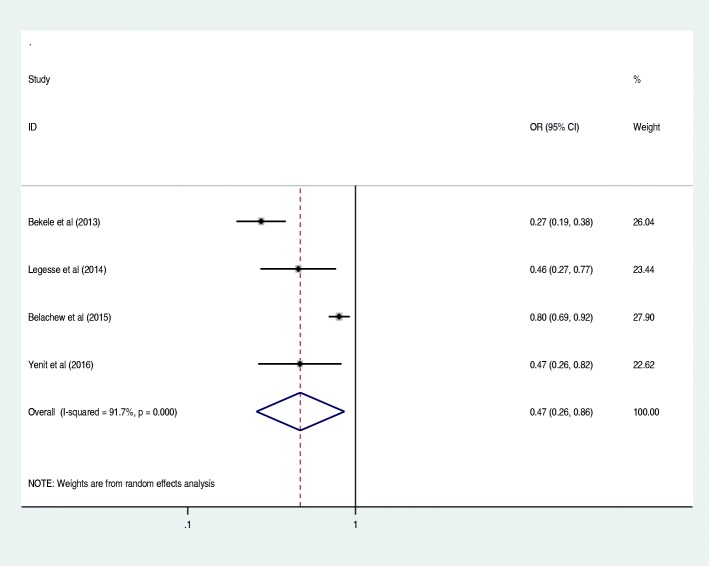
The pooled odds ratio of the association between place of residence and prelacteal feeding

Antenatal care was significantly associated with prelacteal feeding practices in Ethiopia, odds ratio 0.25 (95% CI 0.09, 0.69) (Fig. 3). This indicates that those mothers who had antenatal care for index child 25% times less likely feed prelacteal feeding than counterparts. The odds of developing prelacteal feeding are 3.93 times higher among mothers who was delivered the child at home compared with institutional delivery (OR = 3.93, 95% CI 2.17, 7.10) (Fig. 4). The odds of prelacteal feeding practices are 37% times lesser among mothers consoled on prelacteal feeding during pregnancy than did not consoled (OR = 0.37, 95% CI 0.22, 0.63) (Fig. 5). Timely initiation of breastfeeding 28% times less likely practice prelacteal feeding than those does not initiate breast milk timely within 3 hours (OR = 0.28, 95% CI 0.21, 0.38) (Fig. 6). An urban residence is 47% times less likely practice prelacteal feeding than rural residence (OR = 0.47, 95% CI 0.26, 0.86) (Fig. 7).

## Discussion

This systematic review and meta-analysis was conducted to estimate the pooled prevalence of prelacteal feeding practices and its associated factors among mothers having children age less than 5 years in Ethiopia from 2011 up to 2018.

According to this systematic review and meta-analysis, one fourth (25.29%) of children were given prelacteal foods in Ethiopia. Mothers who had antenatal care for index child, an urban residence and counselling on child feeding practices during pregnancy were less likely to practice prelacteal feeding in Ethiopia. Whereas, mothers who delivered at home gave prelacteal feed than those who delivered in health institutions in Ethiopia.

The prevalence of the prelacteal feeding practice in the current systematic review and meta-analysis is in line with the study conducted in Nepal [3], which it is lower than the study conducted in Nigeria (66.4 and 49.8%) [52], Sub-Saharan African (32.2%) [15] and Vietnam (73.3%) [10]. This disparity may be due to variation of socio-cultural, demographic, and methodological and time of the study across those countries. The magnitude of prelacteal feeding varies across regions in Ethiopia. The highest magnitude was observed in Oromia region followed by Amhara region while the lowest magnitude was observed in southern nation nationalities and peoples of Ethiopia (SNNP). This may be due to cultural difference across regions in Ethiopia. On the other hand, prelacteal feeding practice is decreasing relatively from 2015 and later. This is due to the efforts made by the Ethiopian government and non-government organizations on infant feeding activities during the era of the Millennium Development Goals.

This systematic review and meta-analysis indicated the presence of antenatal care during pregnancy and place of delivery were found to have a statistically significant association with prelacteal feeding practices. Those mothers who had been antenatal care during pregnancy 25% times less likely to practice prelacteal feeding. This study is in line with the study conducted in Sub-Saharan Africa [15] and in Burkina Faso [53]. The possible reason may be during the period antenatal visit, there is counseling on infant feeding practices since an infant and young child feeding (IYCF) strategy is also the component of the national nutrition strategy in Ethiopia which includes those pregnant women. Women who gave birth at their home is 3.93 times more likely to practice prelacteal feeding than who gave birth at health institutions. This finding is consistent with the study done in Sub-Saharan Africa [15], Nigeria trained study [54]**,** Nigeria population based demographic and health survey [55], in Burkina Faso and South Africa [53]. This may be due to the fact that home delivery is attended by traditional birth attendants who do not have the knowledge of exclusive breastfeeding and the harms of prelacteal feeding practice. Moreover, in Ethiopia, those elders and traditional birth attendants promote prelacteal feeding practices. In health facilities, the early initiation of breastfeeding is practiced, the negative effect of prelacteal feeding and the benefit of exclusive breastfeeding are promoted by health professionals during the periods of postnatal stay. Also mothers who give birth in a health facility are likely to be advised by health professionals about the risks associated with prelacteal feeding practices.

Timely initiation of breastfeeding, counseling about infant feeding practice and living in an urban residence had also the positive impact to decrease prelacteal feeding in Ethiopia. Those mothers’ who initiated timely breastfeeding were 28% less likely to practice prelacteal feeding than their counterparts. There is a close relationship between early initiation and avoiding prelacteal feeds. Mothers who received counseling on infant feeding during the perinatal period were less likely to offer prelacteal food than who did not receive counseling. Similar findings was reported in India [56]. This might be due to that counseling is the tool to change the behaviors of mothers to wards prelacteal feeding practice during the time of pregnancy.

Living in an rural residence is 53% times more likely feed prelacteal foods than urban residences in Ethiopia. This is in line with the study done in Nigeria [54, 55]. This might be due to rural residences are relatively having low awareness about the risk of prelacteal feeding and also cultural practices are more common in rural communities.

These studies have certain limitations, which includes articles published in English language, all are cross-sectional articles, and some of the regions did not include because of lack of research.

## Conclusion

In Ethiopia, one in four mothers gave prelacteal foods for their children. Mothers who gave birth at home are more prone to give prelacteal foods. Whereas, antenatal care, timely initiation of breastfeeding, counseling on infant feeding and living in an urban residence decreased prelacteal feeding practices in Ethiopia. On the contrary, home delivery practice increased the risk of prelacteal feeding in Ethiopia. Therefore, the government and health institutions should focus on awareness creation about risk of prelacteal feeding, increase antenatal care service, promote institutional delivery, recommend timely initiation of breastfeeding and increase the counseling service about infant feeding during pregnancies.

## Abbreviations

ANC: Antenatal care
CI: Confidence interval
EBF: Exclusive breastfeeding
IYCF: Infant and young child feeding
OR: Odds ratio
PLF: Prelacteal feeding practice
SNNP: South Nations Nationalities and Peoples
WHO: World Health Organization
